# Arachnoid cyst in the quadrigeminal cistern: A case from Afghanistan

**DOI:** 10.1016/j.radcr.2024.02.048

**Published:** 2024-03-05

**Authors:** Barakatullah Mohammadi, Ahmad Neyazi, Abdul Qadir Qader, Nosaibah Razaqi, Habibah Afzali, Mehrab Neyazi

**Affiliations:** a Department of Neuro & Spine Surgery, Herat Regional Hospital, Herat, Afghanistan; b Afghanistan Center for Epidemiological Studies, Herat, Afghanistan; c Scientific Affairs Department, Herat Regional Hospital, Herat, Afghanistan

**Keywords:** Quadrigeminal arachnoid cyst, Adolescent neurosurgery, Intracranial cysts

## Abstract

Arachnoid cysts, fluid-filled lesions within the central nervous system, pose diagnostic challenges. This study examines a unique case of a quadrigeminal arachnoid cyst in a 13-year-old girl, emphasizing accurate identification and treatment. The patient's symptoms of blurred vision and headaches led to the discovery of papilledema and imaging revealing a sizable cyst causing obstructive hydrocephalus. Urgent surgical intervention involved suboccipital craniectomy and infratentorial-supracerebellar cyst drainage, resulting in favorable postoperative outcomes. Further analysis of anatomical variations, age-related factors, and etiological debates deepens understanding. Diagnostic advancements, notably MRI, are crucial for noninvasive characterization. This case offers nuanced insights into managing arachnoid cysts, highlighting the success of tailored surgical strategies. Recognizing clinical subtleties, utilizing diagnostic innovations, and customizing surgical techniques are essential for navigating complexities. This study underscores the importance of a comprehensive approach in addressing the challenges of arachnoid cysts within the central nervous system.

## Introduction

Arachnoid cysts, characterized as uncommon and benign fluid-filled lesions within the central nervous system, constitute a distinct subset of intracranial abnormalities predominantly containing cerebrospinal fluid (CSF) [1]. These cysts manifest histologically with the encapsulation by normal layers of the arachnoid membrane, notably devoid of any connection with the ventricular system [2]. Despite their infrequent occurrence, arachnoid cysts account for a mere 1% of all intracranial space-occupying lesions [3]. Notably, their development does not typically coincide with anomalous alterations in brain structure [3].

The clinical presentation of arachnoid cysts is highly variable and hinges on factors such as the cyst's location, size, and the age of the affected individual [4]. Among the various brain regions susceptible to the growth of these cysts, the middle fossa region emerges as a prominent site [5]. Quadrigeminal arachnoid cysts, a particularly rare subtype, specifically localize to the quadrigeminal cistern within the brain [6]. Diagnosis of these cysts is typically achieved through imaging modalities such as computed tomography (CT) or magnetic resonance imaging (MRI). The primary therapeutic approach for quadrigeminal arachnoid cysts is surgical intervention, underscoring the limited spectrum of available treatment options [7]. In this context, this case report explores the unique clinical presentation, diagnostic challenges, and surgical management of a quadrigeminal arachnoid cyst, shedding light on the complexities associated with this rare neurological entity.

The work has been reported in line with the SCARE 2020 criteria [8].

## Case presentation

**Fig. 1:**
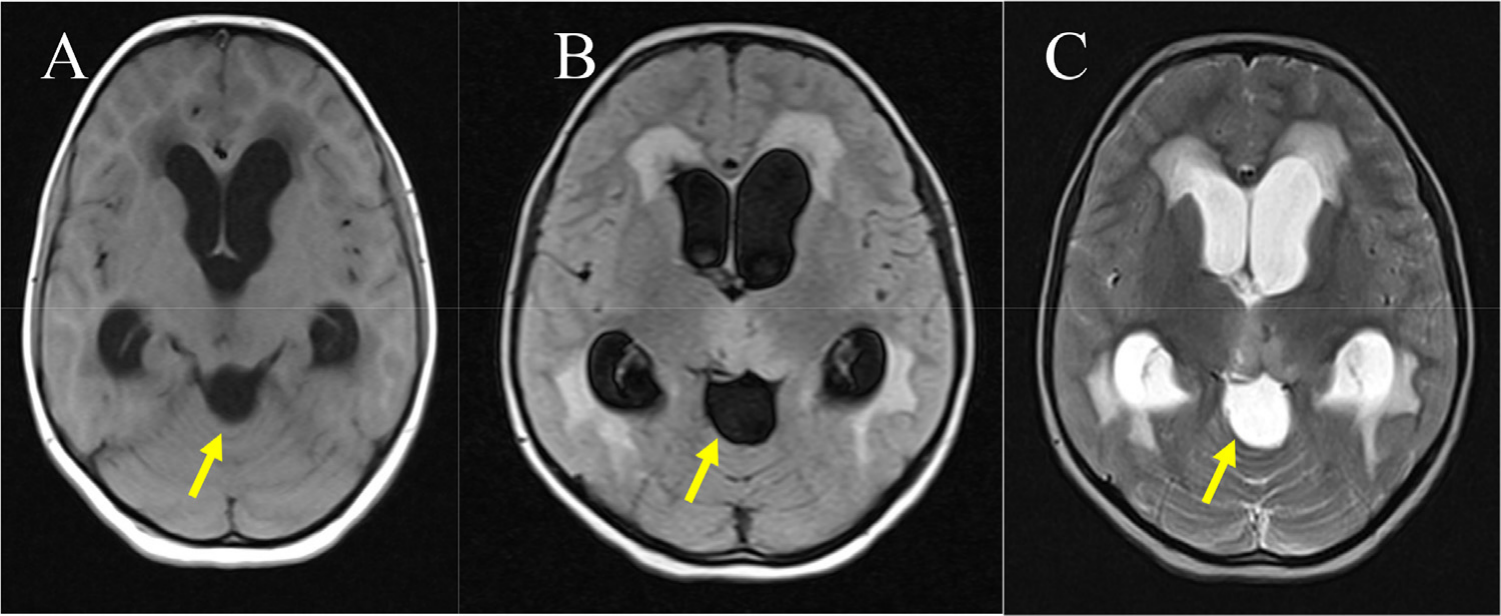
Noncontrast Axial MRI shows a well-demarcated, homogenously cystic mass (arrows) occupying the quadrigeminal cistern and following cerebrospinal fluid (CSF) signal intensity on T1-weighted (A), FLAIR (B), and T2-weighted images (C).

**Fig. 2:**
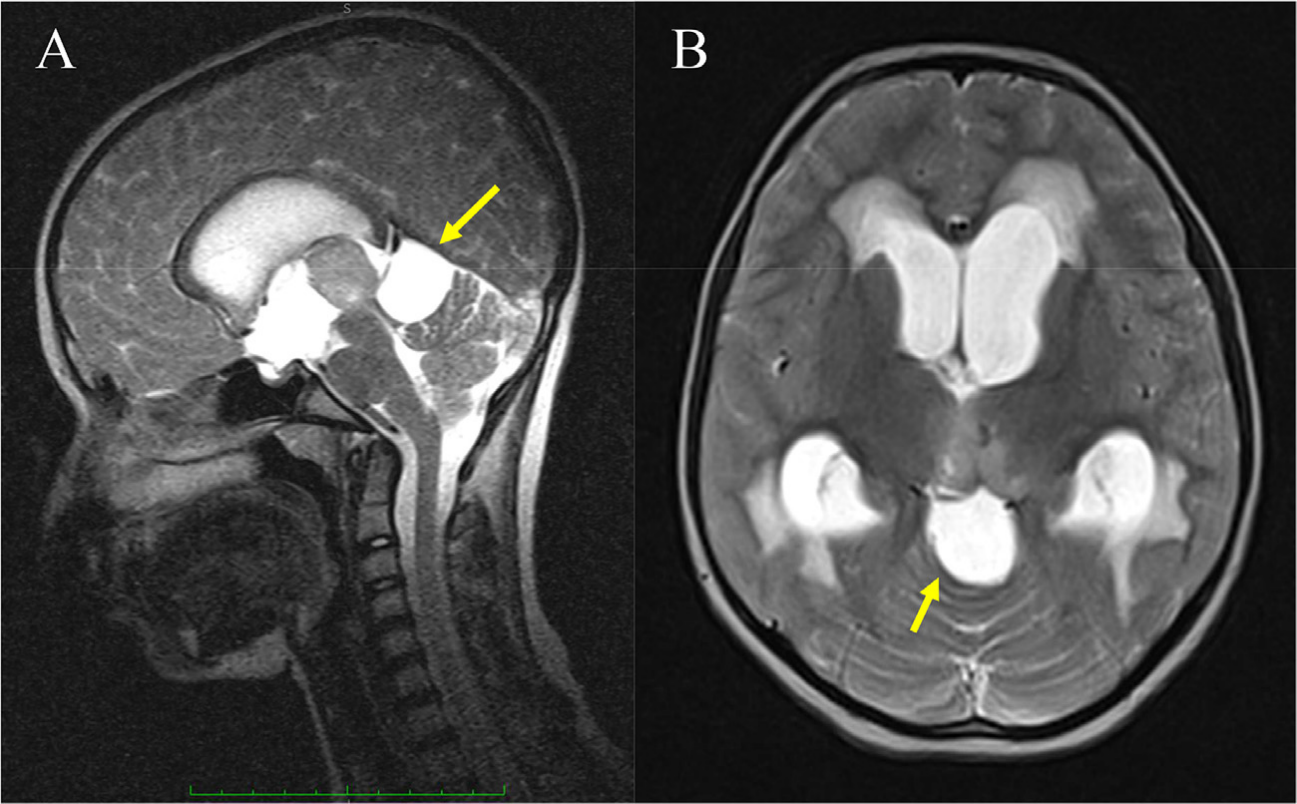
Sagittal (A) and Axial (B) T2W MRI image demonstrated enlargement of the lateral and third ventricular system secondary to quadrigeminal cistern arachnoid cyst compressing the brainstem, cerebellum and aqueduct of sylvius is obstructed and occupying quadrigeminal plate cistern causing hydrocephalus.

**Fig. 3:**
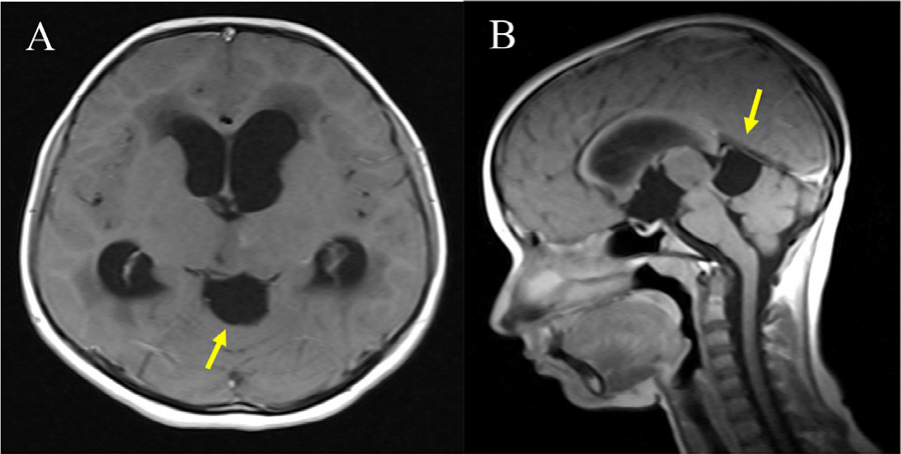
Axial (A) and Sagittal T1 (B) post contrast MRI showing no enhancement of cyst walls and no mural nodule on post Gadolinium administration.

A 13-year-old female student sought medical attention reporting a three-month duration of blurred vision and persistent dull occipital headaches. Subsequent evaluation by an ophthalmologist identified papilledema. Upon admission, neurological examinations indicated an absence of abnormalities, except for the presence of papilledema observed in both ocular fundi. The magnetic resonance imaging (MRI) results revealed a cystic space-occupying lesion measuring 2.7 × 2.8 × 3.1 cm in the quadrigeminal region. The lesion exhibited signal characteristics consistent with cerebrospinal fluid, displaying high signal intensity on T2-weighted images and low signal intensity on T1-weighted and FLAIR images (Fig. 1). The cyst exerted mass effect onto the aqueduct, leading to dilatation of the lateral and third ventricles, Overall, these findings are consistent with a diagnosis of a quadrigeminal region arachnoid cyst with associated obstructive hydrocephalus. (Fig. 2). Following Gadolinium administration exhibited no wall or peripheral nodules enhancement or enhanced solid component. (Fig. 3).

The patient underwent a surgical procedure with placement in the sitting position, utilizing a suboccipital craniectomy, and an infratentorial-supracerebellar approach to access the quadrigeminal region. Within this region, a substantial cyst, characterized by a relatively thick and opaque membrane, was readily exposed. Notably, the cyst extended upward beyond the free edge of the tentorium. During exploration, the precentral cerebellar vein was identified coursing along the dorsal aspect of the cyst wall in the midline. Subsequent to carefully opening the cyst wall, a significant volume of crystal-clear fluid, resembling cerebrospinal fluid (CSF), was observed to emanate. Efforts were undertaken to strip off as much of the cyst wall as possible, facilitating unimpeded communication with the ambient cistern. The bilateral basal vein of Rosenthal was visualized prominently. Importantly, postprocedural examination revealed an absence of any space-occupying mass between the precentral cerebellar vein and the veins of Rosenthal. Following this intervention, the patient underwent a ventriculoperitoneal shunt placement. The patient's postoperative course has demonstrated favorable outcomes, affirming the efficacy of the employed surgical strategy in the management of quadrigeminal arachnoid cysts. This observation contributes to the growing body of evidence supporting the positive impact of targeted interventions on patient recovery in such cases.

## Discussion

Within the spectrum of intracranial arachnoid cysts, a notable prevalence exists above the tentorium, with infratentorial arachnoid cysts representing a comparatively rarer manifestation. Infratentorial arachnoid cysts are categorized into 5 distinct locations: (1) midline retrocerebellar, (2) lateral (or hemispheric) retrocerebellar, (3) cerebellopontine angle, (4) clival, and (5) quadrigeminal. Among these, retrocerebellar and cerebellopontine angle cysts emerge as the more frequently encountered subtypes [9]. This distribution underscores the nuanced anatomical variations in the presentation of arachnoid cysts within the infratentorial compartment, with implications for both diagnostic considerations and surgical approaches.

Furthermore, a notable distinction can be drawn based on the age at clinical presentation, contributing to the intricate clinical profile of infratentorial arachnoid cysts. Quadrigeminal cysts, specifically, tend to manifest in infants or young children. In contrast, the 2 variations of retrocerebellar cysts are predominantly observed in adolescents or young adults. Notably, cerebellopontine angle cysts exhibit a distinct age predilection, being encountered almost exclusively in the adult population [9,10]. This age-related distribution underscores the importance of considering age as a key factor in the differential diagnosis of infratentorial arachnoid cysts, influencing both the clinical presentation and the selection of appropriate diagnostic and therapeutic strategies.

Considerable controversy surrounds the etiological underpinnings of arachnoid cysts, as multiple theories, encompassing both acquired and developmental perspectives, vie for explanatory dominance. Acquired lesions are attributed to meningeal inflammation and trauma, whereas defective duplication of the leptomeninges and the growth of ectopic ependymal and arachnoid tissue are posited as developmental mechanisms [9]. Predominantly, it is consensually acknowledged that most intracranial arachnoid cysts, particularly those beneath the tentorium cerebelli, are of developmental origin. Additionally, there is a proposition that certain arachnoid cysts may evolve from embryonic rests, metamorphosing into rudimentary secretory organs or mature choroid plexus structures [9,11,12]. The occurrence of an ectopic choroid plexus within a posterior fossa cyst is a rarity, with the majority observed in retrocerebellar lesions; only one documented case previously involved a quadrigeminal plate cyst [13].

The delineation between intra-arachnoid, subarachnoid, or subdural localization of such cysts may pose challenges during surgical intervention [9]. However, electron microscopy studies have elucidated that cysts within the quadrigeminal plate cistern are situated within the arachnoid, forming through a process of splitting or duplication of the arachnoid membrane [11,14]. This intricate understanding of cyst microanatomy holds significance in guiding surgical decision-making and underscores the necessity for a nuanced comprehension of the varied etiologies underpinning arachnoid cyst development.

Quadrigeminal arachnoid cysts exhibit a notable anatomical variation, with approximately 25% of cases demonstrating extension above the tentorial notch. In instances where the supratentorial component attains exceptional size, an intriguing observation emerges wherein the tentorium cerebelli may be depressed rather than elevated [15]. This distinctive characteristic underscores the dynamic nature of quadrigeminal arachnoid cysts and highlights the potential for diverse structural interactions within the intracranial compartment. Clinicians and surgeons should be cognizant of these anatomical nuances, as they can have implications for diagnostic interpretation and surgical planning when confronted with cases of quadrigeminal arachnoid cysts presenting with supratentorial extension [15].

Motor signs, such as hemiparesis [16], lower extremity weakness [10], or generalized spasticity and clonus [15], are exceptionally rare in the context of quadrigeminal arachnoid cysts. Lateral rectus palsy [15], [16], [17], though reported occasionally, is considered a potentially "false" localizing sign associated with increased intracranial pressure. The remarkable variability in clinical presentations underscores the complexity of the pathophysiology associated with quadrigeminal arachnoid cysts and emphasizes the need for a comprehensive understanding of the diverse neurological manifestations that may arise in affected individuals.

Historically, diagnosing arachnoid cysts posed challenges, with diagnostic ventriculography even leading to cerebral herniation in some cases [11]. However, the advent of imaging modalities such as CT scans and MRI has significantly enhanced the diagnostic capabilities for intracranial arachnoid cysts, leading to a surge in reported cases. In particular, the increased utilization of MRI has facilitated the detection of arachnoid cysts in the quadrigeminal cistern, even during their asymptomatic stages, consequently leading to a higher prevalence in adult cases. MRI scans offer superior insights into the location and size of the cyst, and crucially, they provide clear discrimination between communicating and noncommunicating types. Notably, delayed or sequential MRI studies have occasionally revealed slow filling and delayed clearance of contrast medium within the cyst [18], [19]. This noninvasive imaging approach holds significant promise in elucidating cerebrospinal fluid kinetics and unraveling the natural history of these intricate leptomeningeal cysts. Furthermore, it stands to contribute substantially to the establishment of a precise definition and classification for true arachnoid cysts, enhancing our understanding of their characteristics and evolution.

## Conclusion

This case report elucidates the intricate clinical presentation, diagnostic challenges, and successful surgical management of a quadrigeminal arachnoid cyst in an adolescent patient. The distinctive features, including papilledema, characteristic imaging findings, and intraoperative observations, contribute to our understanding of the complexities associated with this rare neurological entity. The case underscores the importance of tailored surgical approaches guided by anatomical precision and highlights the potential benefits of ventriculoperitoneal shunting. Overall, this case contributes valuable insights for clinicians managing quadrigeminal arachnoid cysts and advances our understanding of their clinical nuances. Postoperatively, the patient exhibited favorable outcomes, underscoring the success of the surgical approach for quadrigeminal arachnoid cysts.

## Ethical approval

This study was approved by the Scientific council of the Herat Regional Hospital (approval number: #23.1.078) on 12 September 2023.

## Author's contribution

All authors contributed equally.

## Patient consent

Written informed consent was obtained from the patient's parents/legal guardian for publication and any accompanying images. A copy of the written consent is available for review by the Editor-in-Chief of this journal on request.
